# Causal association between B cell count and psoriasis using two‐sample Mendelian randomization

**DOI:** 10.1111/jcmm.70089

**Published:** 2024-09-11

**Authors:** Zongfeng Zhao, Jie Cheng, Jian Zhu, Sheng Lu, Hongli Lv, Xiujuan Wu

**Affiliations:** ^1^ Central Laboratory Shanghai Xuhui Central Hospital Shanghai China; ^2^ Department of Urology Shanghai Xuhui Central Hospital Shanghai China; ^3^ Department of Dermatology Shanghai Xuhui Central Hospital Shanghai China; ^4^ Department of Dermatology Jia Ding Central Hospital Shanghai China

**Keywords:** B cell count, Mendelian randomization, psoriasis

## Abstract

To investigate the causality between B cell count and psoriasis by Mendelian randomization (MR). Collected B cell count and psoriasis data from IEU Open GWAS Project. Employed inverse variance weighting (IVW), MR‐Egger, WM, weighted mode for analysis, ensuring result robustness. Assessed horizontal pleiotropy with MR‐Egger, detected outliers using MR‐PRESSO and examined instrumental variables heterogeneity with Cochran's *Q*‐test. The IVW method suggested an association between a genetically predicted memory B cell count and the risk of psoriasis vulgaris. IVW results also showed no causality between other exposure factors and the corresponding outcomes. Also, the global test of MR‐PRESSO analysis showed a significant association between a genetically predicted transitional absolute B cell count and the lower risk of psoriasis vulgaris. MR‐Egger regression showed that horizontal pleiotropy did not influence the analysis results. We found that memory B cell absolute counts are associated with a lower risk of psoriasis. These data further elucidate the role of memory B cells in psoriasis and provide new options for psoriasis treatment.

## 1 INTRODUCTION

Psoriasis is a chronic immune disease that causes a rash with itchy, scaly patches, most commonly on the elbows, knees, scalp, and trunk. It can occur at any age and greatly affects people's quality of life.^1^ The prevalence of psoriasis in adults ranges between 0.27% and 11.4%, with sex, geography, age, genetics, ethnicity and environmental factors contributing to the variations in the prevalence of the disease.^2^ The management currently involves addressing the physical and psychosocial aspects of the disease; however, psoriasis treatment is still limited, mainly due to unclear pathophysiology.

Pathophysiology of psoriasis involves infiltration of the skin by activated T cells, which stimulate the proliferation of keratinocytes and form thick plaques.^3^ Furthermore, recent studies have discovered that B cells may contribute to the exacerbation of psoriasis. B cells regulate immune responses positively and negatively; they have multiple functions essential for immune homeostasis, such as T cell activation, antigen presentation and cytokine production.^4^ Mizumaki et al.^5^ investigated the role of regulatory B cells in a mice model of psoriasis and found that increased cells suppress interleukin (IL)‐23‐mediated psoriasis‐like inflammation through Treg (regulatory T cells) expansion and inhibition of Th17 (T helper 17 cells) differentiation. Moreover, Kahlert et al.^6^ performed a systematic longitudinal analysis of B‐cell subpopulations and corresponding immunoglobulin (Ig) levels to investigate the potential impact of B cells in psoriasis, finding the alteration of B‐cell subsets, particularly up‐regulation of transitional B cells (also known as TrB cells), short‐lived plasmablasts (PB), and IgA might be part of an inflammation‐induced compensatory mechanism in psoriasis. Nevertheless, these studies contain reverse causality and confounding factors.

Mendelian randomization (MR) is a new method based on Mendel's laws of inheritance and instrumental variable estimation methods that use genetic variants to assess causal relationships and how modifiable exposures influence different outcomes.^7^ A genetic variant can be considered as an instrumental variable for a given exposure if it satisfies the instrumental variable assumptions^1^, ^8^: it does not affect the outcome^2^; it is not associated with the outcome due to confounding pathways^3^; it is associated with the exposure. In recent years, MR has been increasingly applied to explore the repurposing potential of available drugs.^9^, ^10^, ^11^ For example, MR studies recently discovered that body mass index (BMI), blood lipids, unsaturated fatty acids, smoking, and drinking promote psoriasis.^12^, ^13^, ^14^, ^15^ However, the relationship between B cell count and psoriasis using the MR method has not yet been tested.

In this study, we applied an MR analysis to investigate whether B cell count can be used as a predictive factor for psoriasis, which should be further explored through MR analysis.

## 2 MATERIALS AND METHODS

### 2.1 Study design

**FIGURE 1:**
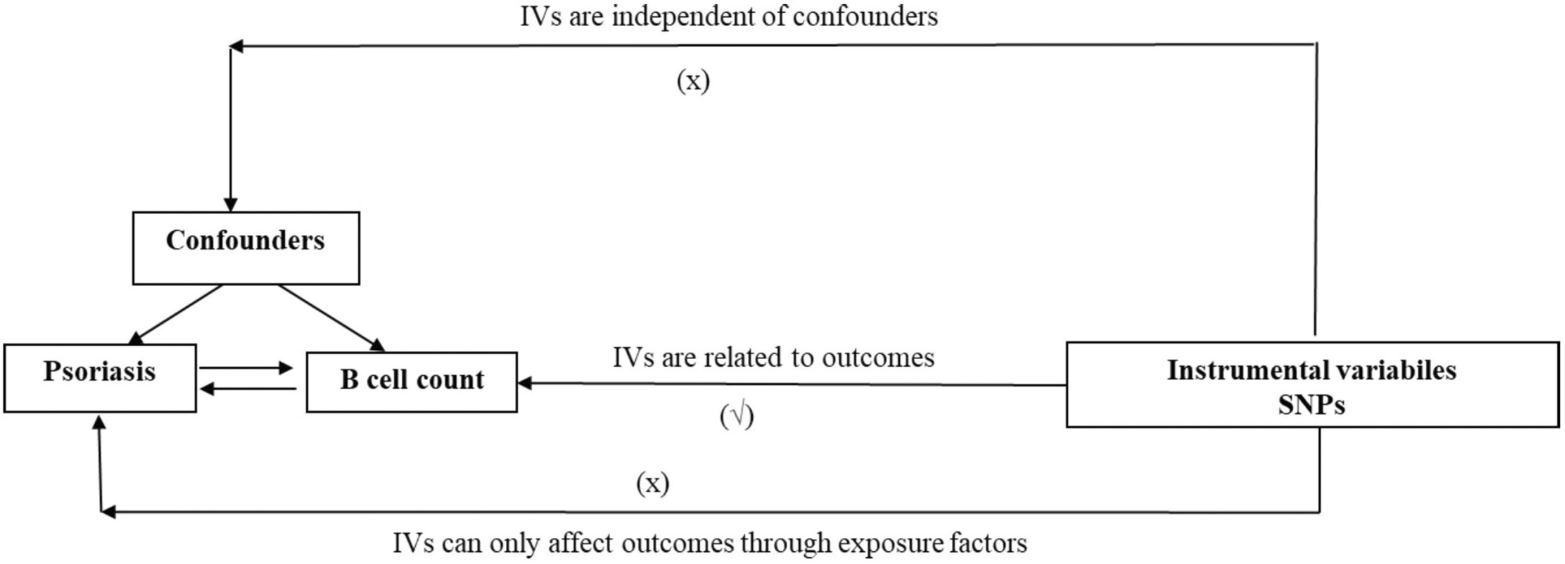
Overview of Mendelian randomization. Single nucleotide polymorphisms (SNPs); inverse‐variance weighted (IVW); weighted median (WM); leave‐one‐out (LOO).

The analysis adhered to three assumptions of MR studies (Figure 1).^8^ Open‐source data used in this study did not require ethical approval. Also, GWAS data has been ethically compliant with the Helsinki Declaration.

### 2.2 Data sources

Summary data from available genome‐wide association studies (GWAS) were employed to perform the analyses. The study did not require approval from the ethics committee. Psoriasis data were sourced from the ukb‐a‐100 database (‘Non‐cancer illness code self‐reported: psoriasis’; https://gwas.mrcieu.ac.uk/datasets/ukb‐a‐100/) that included 3,871 case samples, 333,288 control samples, and 10,894,596 single‐nucleotide polymorphisms (SNPs) and finn‐b‐PSORI_STRICT databases (‘Psoriasis (vulgaris), strict definition’; https://gwas.mrcieu.ac.uk/datasets/finn‐b‐PSORI_STRICT/) that included 334 case samples, 212,242 control samples and 16,380,456 SNPs. Data on B cell count was sourced from: data from absolute B cells were from the ebi‐a‐GCST90001642 database (https://gwas.mrcieu.ac.uk/datasets/ebi‐a‐GCST90001642/) that included 3,653 samples and 15,195,758 SNPs; data from memory B cell count were from the ebi‐a‐GCST90001407 database (https://gwas.mrcieu.ac.uk/datasets/ebi‐a‐GCST90001407/) that included 3,656 samples and 15,048,937 SNPs; data from switched memory B cell count were from the ebi‐a‐GCST90001403 database (https://gwas.mrcieu.ac.uk/datasets/ebi‐a‐GCST90001403/) that included 3,656 samples and 15,048,937 SNPs; data from transitional absolute B cell count were from the ebi‐a‐GCST90001577 database (https://gwas.mrcieu.ac.uk/datasets/ebi‐a‐GCST90001577/) that included 3,656 samples and 15,048,937 SNPs; data from un‐switched memory B cell count were from the ebi‐a‐GCST90001398 database (https://gwas.mrcieu.ac.uk/datasets/ebi‐a‐GCST90001398/) that included 3,656 and 15,048,937 SNPs.

### 2.3 Selection of instrumental variables

MR studies used SNPs as instrumental variables (IVs). The IVs included in this study met the following criteria^1^, ^16^: SNPs associated with B cell count were screened based on the following values: *p* < 5 × 10^−6^. Then, SNPs with a minimum minor allele frequency (MAF) >0.01 were screened, and SNPs with linkage disequilibrium *R* ^2^ < 0.001 or window size = 10,000 kb were excluded to avoid confounding effects. When the selected IVs are not present in the summary data of the outcome, SNPs with high LD (*R* ^2^ > 0.8) with the IV as proxy SNPs for replacement were screened. Next, the *F*‐value for each SNP in the IV was calculated to assess IV strength, excluding potential weak instrument bias between the IV and exposure factors, using the following formula: *F = R* ^2^ × (*N*‐2)/(*1*‐*R* ^2^), where *R* ^2^ represents the proportion of exposure variance explained by the SNP in the IV. The requirement for the *F*‐value is >10.

### 2.4 Mendelian randomization and sensitivity analysis

The MR analysis followed the Strengthening the Reporting of Observational Studies in Epidemiology using Mendelian Randomization (STROBE‐MR) guidelines.^17^ The analysis process adhered to three assumptions of MR studies (Figure 1).^8^ Various methods were used to estimate the causal relationship between the exposure and outcome, including weighted median (WM), inverse variance weighting (IVW), simple model, and MR‐Egger, weighted model. This analysis primarily employed the IVW method to evaluate the causal relationship between exposure and outcome risk by calculating the odds ratio (OR) and 95% confidence interval (CI).^18^ All analyses were performed using R 4.1.2 software, incorporating packages such as TwoSampleMR and MR‐PRESSO. Heterogeneity was tested using Cochran's *Q*, and pleiotropy was examined through MR‐Egger regression of intercept values.Scatter plots and sensitivity analysis plots were used for visualization.^19^ As there are four outcome factors in this study, the false discovery rate (FDR) correction method was applied to correct for multiple testing, with PFDR <0.05 considered statistically significant. Scatter plots and sensitivity analysis plots were used for visualization.

## 3 RESULTS

### 3.1 Inclusion of instrumental variables

After ensuring the robustness, heterogeneity, and generalizability of SNPs while avoiding their potential confounding effects and pleiotropy, 17, 11, 16, 21 and 13 IVs related to the absolute B cell count, memory B cell count, switched memory B cell count, transitional absolute B cell count, and un‐switched memory B cell count were selected. After calculation, the mean *F*‐statistic value of the IVs was 25.22, 24.55, 23.40, 23.75 and 24.91, with a minimum value of 20.99, 21.61, 21.04, 20.90 and 21.24, and a maximum value of 40.85, 42.24, 29.05, 33.13 and 33.29 for an absolute count of B cells, memory B cell count, switched memory B cell count, transitional absolute B cell count and un‐switched memory B cell count, respectively.

### 3.2 The causal effect of B cell count on psoriasis

**FIGURE 2:**
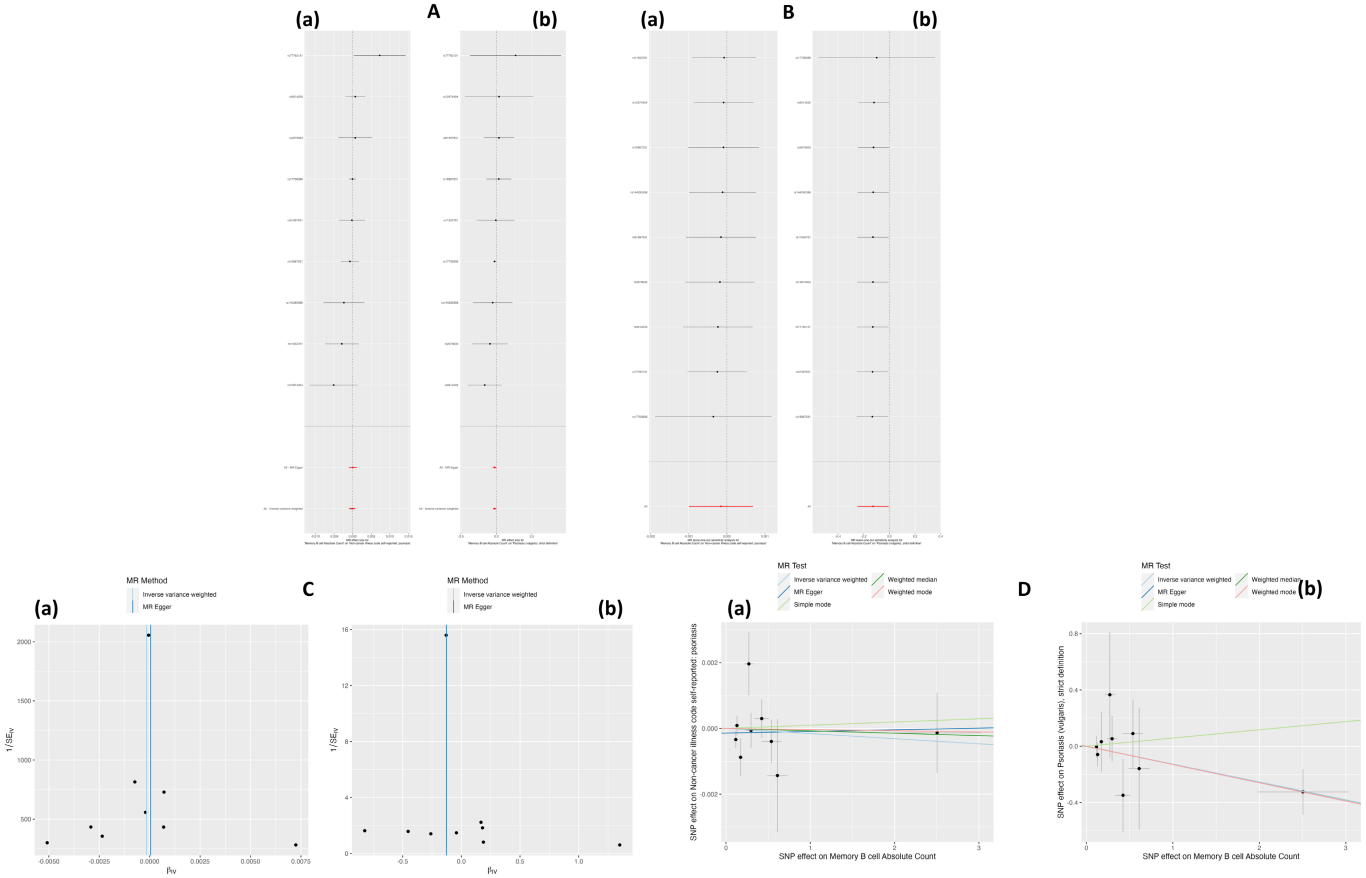
A causal association between memory B cell absolute count and the risk of psoriasis vulgaris. Forest plot image (A); leave‐one‐out plot (B); funnel plot image (C); scatter plot image (D). (a) Memory B cell absolute count versus ‘non‐cancer illness code self‐reported: Psoriasis’; (b) memory B cell absolute count versus ‘Psoriasis (vulgaris), strict definition’.

The association between genetically predicted absolute B cell count, memory B cell count, switched memory B cell count, transitional absolute B cell count and un‐switched memory B cell count and the risk of psoriasis, plaque psoriasis is shown in Table 1. IVW method suggested an association between a genetically predicted memory B cell count and the risk of psoriasis vulgaris [OR (95% CI): 0.88 (0.78–0.99), *p* = 0.04] (see Table 1, Figure 2). IVW results showed no causality between other exposure factors and the corresponding outcomes (see Table 1, Figures [Link], [Link], [Link], [Link]).

**TABLE 1:** Association between genetically predicted B cell count (absolute count of B cells, memory B cell count, switched memory B cell count, transitional absolute B cell count and un‐switched memory B cell count) and the risk of psoriasis, plaque psoriasis.

Exposure	Outcome	N.SNPs	Methods	OR (95% CI)	*p*
Absolute B cell count	Non‐cancer illness code self‐reported: psoriasis	16	IVM	1.00 (1.00–1.00)	0.33
			MR Egger	1.00 (1.00–1.00)	0.94
			WM	1.00 (1.00–1.00)	0.88
			Simple mode	1.00 (1.00–1.00)	0.78
			Weighted mode	1.00 (1.00–1.00)	0.93
			MR‐PRESSO	1.00 (1.00–1.00)	0.17
	Psoriasis (vulgaris), strict definition	15	IVM	1.06 (0.81–1.38)	0.69
			MR Egger	1.10 (0.70–1.74)	0.67
			WM	1.11 (0.77–1.61)	0.58
			Simple mode	1.32 (0.75–2.35)	0.35
			Weighted mode	1.13 (0.74–1.72)	0.58
			MR‐PRESSO	1.06 (0.81–1.38)	0.38
Memory B cell absolute count	Non‐cancer illness code self‐reported: psoriasis	9	IVM	1.00 (1.00–1.00)	0.72
			MR Egger	1.00 (1.00–1.00)	0.93
			WM	1.00 (1.00–1.00)	0.88
			Simple mode	1.00 (1.00–1.00)	0.91
			Weighted mode	1.00 (1.00–1.00)	0.94
			MR‐PRESSO	1.00 (1.00–1.00)	0.72
	Psoriasis (vulgaris), strict definition	9	IVM	0.88 (0.78–0.99)	**0.04**
			MR Egger	0.88 (0.77–1.01)	0.12
			WM	0.88 (0.76–1.02)	0.09
			Simple mode	1.06 (0.63–1.78)	0.83
			Weighted mode	0.88 (0.77–1.00)	0.09
			MR‐PRESSO	0.88 (0.78–0.99)	0.01
Switched memory B cell absolute count	Non‐cancer illness code self‐reported: psoriasis	14	IVM	1.00 (1.00–1.00)	0.29
			MR Egger	1.00 (1.00–1.00)	0.76
			WM	1.00 (1.00–1.00)	0.92
			Simple mode	1.00 (1.00–1.00)	0.74
			Weighted mode	1.00 (1.00–1.00)	0.78
			MR‐PRESSO	1.00 (1.00–1.00)	0.39
	Psoriasis (vulgaris), strict definition	15	IVM	1.21 (0.94–1.54)	0.14
			MR Egger	1.12 (0.79–1.58)	0.54
			WM	1.11 (0.79–1.55)	0.54
			Simple mode	0.92 (0.55–1.53)	0.74
			Weighted mode	1.07 (0.78–1.46)	0.7
			MR‐PRESSO	1.21 (0.94–1.54)	0.16
Transitional B cell absolute count	Non‐cancer illness code self‐reported: psoriasis	19	IVM	1.00 (1.00–1.00)	0.52
			MR Egger	1.00 (1.00–1.00)	0.72
			WM	1.00 (1.00–1.00)	0.91
			Simple mode	1.00 (1.00–1.00)	0.58
			Weighted mode	1.00 (1.00–1.00)	0.98
			MR‐PRESSO	1.00 (1.00–1.00)	0.53
	Psoriasis (vulgaris), strict definition	20	IVM	0.82 (0.65–1.03)	0.09
			MR Egger	0.84 (0.60–1.17)	0.31
			WM	0.77 (0.56–1.08)	0.13
			Simple mode	0.55 (0.30–1.00)	0.07
			Weighted mode	0.75 (0.55–1.02)	0.08
			MR‐PRESSO	0.82 (0.65–1.03)	**0.03**
Un‐switched memory B cell absolute count	Non‐cancer illness code self‐reported: psoriasis	10	IVM	1.00 (1.00–1.00)	0.82
			MR Egger	1.00 (0.99–1.00)	0.49
			WM	1.00 (1.00–1.00)	0.78
			Simple mode	1.00 (0.99–1.00)	0.48
			Weighted mode	1.00 (1.00–1.00)	0.85
			MR‐PRESSO	1.00 (1.00–1.00)	0.66
	Psoriasis (vulgaris), strict definition	11	IVM	1.01 (0.66–1.56)	0.95
			MR Egger	1.39 (0.47–4.14)	0.57
			WM	0.94 (0.51–1.73)	0.84
			Simple mode	0.99 (0.40–2.49)	0.99
			Weighted mode	0.95 (0.41–2.18)	0.91
			MR‐PRESSO	1.01 (0.66–1.56)	0.94

Abbreviations: IVW, inverse variance weighting; MR, Mendelian randomization; WM, weighted median.

Results from MR‐Egger regression showed that horizontal pleiotropy did not influence the analysis results; no outliers were observed (Table 2), which suggests that this data is robust.

**TABLE 2:** Results of IV heterogeneity test and multiplicity test.

Exposure	Outcome	Heterogeneity		Pleiotropy	
		*Q* statistic (IVW)	*p‐*value	MR‐Egger intercept	*p‐*value
B cell absolute count	Non‐cancer illness code self‐reported: psoriasis	12.32	0.65	−0.001	0.427
	Psoriasis (vulgaris), strict definition	7.22	0.93	−0.011	0.811
Memory B cell absolute count	Non‐cancer illness code self‐reported: psoriasis	9.38	0.31	−0.001	0.545
	Psoriasis (vulgaris), strict definition	3.22	0.92	−0.001	0.992
Switched memory B cell absolute count	Non‐cancer illness code self‐reported: psoriasis	18.65	0.13	−0.001	0.254
	Psoriasis (vulgaris), strict definition	14.14	0.44	0.026	0.542
Transitional B cell absolute count	Non‐cancer illness code self‐reported: psoriasis	20.1	0.33	−0.001	0.973
	Psoriasis (vulgaris), strict definition	10.4	0.92	−0.008	0.857
Unswitched memory B cell absolute count	Non‐cancer illness code self‐reported: psoriasis	18.03	0.03	0.001	0.408
	Psoriasis (vulgaris), strict definition	7.29	0.7	−0.055	0.555

## 4 DISCUSSION

B cells are a key player in the adaptive immune response and are responsible for humoral immunity in mammals. These immune cells can promote inflammatory processes by producing cytokines that act on immune cells that mediate inflammation.^20^ Dysregulation of B‐cell function can lead to severe consequences for the host, and different B‐cell malignancies have been described so far^21^, ^22^; still, B cells have largely been neglected in studies of psoriasis, potentially owing to their limited presence in involved psoriatic skin. Only a few studies have found that B cell numbers are elevated in lesional skin relative to control skin specimens in several inflammatory diseases,^23^ including psoriasis.^6^, ^24^, ^25^, ^26^ However, most of these studies were retrospective and observational studies that did not consider confounding factors such as possible genetic influence on disease development. This study investigated the causality between B cell count and psoriasis using MR, a method that applies to the use of genetic variation to address causal questions about how modifiable exposures influence different outcomes. We found that memory B cell count is associated with the lower risk of psoriasis. These data shed new light on the role of B cells in psoriasis and open new ways for psoriasis treatment.

Mature B cells recirculate through the blood and accumulate in the follicles of secondary lymphoid tissues. After stimulation with their cognate (self‐) antigen, mature B cells eventually form short‐lived PB, memory B cells or long‐lived plasma cells (PC). Memory B cells, generated during T‐dependent and T‐independent responses to the respective antigen, provide a rapid response to subsequent autoantigen exposure.^27^ Previous studies have confirmed that memory B cells are formed under inflammatory conditions, such as during flare‐ups of autoimmune diseases (ADs). These memory B cells can migrate into inflammatory tissues, where they are relatively protected from therapeutic interventions.^28^ For example, in patients with multiple sclerosis (MS), memory B cells are a major source of lymphotoxin and TNF‐α.^29^ Additionally, a trial of atacicept in patients with rheumatoid arthritis (RA) observed a transient increase in memory B cells.^30^ In systemic lupus erythematosus (SLE), treatment with tocilizumab led to a reduction in both memory B cells and Ig levels, indicating a decrease in plasma cell numbers.^31^ Some recent data have shown that memory B cells localize to healthy skin of humans and other mammalian species with likely homeostatic functions in host defence, regulating microbial communities and wound healing.^23^ Yet the association between memory B cells and psoriasis has rarely been reported and remains unfully understood. A study from 2018^6^ that investigated aberrant B‐cell subsets and Ig levels in 34 patients (18 males and 16 females, mean age 50.6 ± 15.4 years) with moderate‐to‐severe psoriasis found that memory B cells and total PC were significantly lower in patients with psoriasis. These reports highlight the significant role of memory B cells in the pathogenesis of ADs and suggest their potential for treatment.

In 2000, Ochsenbein et al.^27^ identified differentially expressed genes with altered expression in psoriasis lesions (*n* = 216 patients) and found several genes associated with B cells. In this study, we selected 11 SNPs to analyse the association between memory B cell count and psoriasis vulgaris. This is the first study that suggests a genetic effect of memory B cell count on the risk of psoriasis vulgaris. Considering that stimulated memory B cells contribute to inflammation by cytokine expression and antigen presentation to T cells in several cutaneous ADs,^32^ we suspect this type of cells may be involved in psoriasis. Previous studies utilizing MR have shown that immune cells, particularly memory B cells, play a significant role in the risk of systemic ADs, such as Sjögren's syndrome (SS).^33^ This finding implies that psoriasis and other ADs may share a common signalling pathway associated with memory B cell counts. However, further clinical data are necessary to confirm these results.

This study found no association between other B cell subsets and psoriasis. However, some observational studies reported different findings that might be taken into consideration. For example, Yanaba et al.^24^ identified the contribution of B cells to the pathogenesis of psoriasis and found direct evidence that regulatory B cell subset cells, a type of B cell subset that suppress immunopathology by prohibiting the expansion of pathogenic T cells and other pro‐inflammatory lymphocytes, regulate imiquimod‐induced skin inflammation and offer insights into regulatory B cell‐based therapies for the treatment of psoriasis. In addition, transitional B cells are assumed to play a role in ADs, such as SLE and psoriasis.^25^ More recently, Kahlert and his team^6^ found that alteration of B‐cell subsets, particularly up‐regulation of transitional B cells, might be part of an inflammation‐induced compensatory mechanism in psoriasis. A significant elevation of these cells seems to be controlled by NFATc1, a transcription factor that controls the activity of IL‐10 production in psoriasis‐like skin inflammation.^34^ Furthermore, the multi‐stage differentiation of B cells and the specific markers for IL‐10‐producing B cells remain inadequately defined.^35^ Additional research is necessary to elucidate the role of B cells in the pathogenesis of psoriasis.

This study represents the first evaluation of the association between B cell count and psoriasis. Our study's strengths include the use of a rigorous MR design, multiple sensitivity analyses and large‐scale GWAS data. Limitations include the potential for unmeasured pleiotropy, residual confounding and the reliance on predominantly European ancestry populations, limiting generalizability. Future research could benefit from more diverse ethnic samples, functional validation of implicated genetic variants and exploration of potential mediating pathways linking B cell count to psoriasis.

In conclusion, the present MR study did demonstrate that transitional absolute B cell count and memory B cell count are associated with psoriasis. Revealing the potential association of B cell count with the occurrence of psoriasis, which can provide clues for further studies of psoriasis diagnosis, prevention and treatment strategies. Also, further enrolling a bigger population to study the function of B cell count in the pathogenesis of psoriasis is warrant.

## AUTHOR CONTRIBUTIONS

**Zongfeng Zhao:** Formal analysis (lead); writing – original draft (equal); writing – review and editing (equal). **Jie Cheng:** Resources (lead); writing – original draft (equal); writing – review and editing (equal). **Jian Zhu:** Data curation (equal); investigation (equal); methodology (equal); writing – original draft (equal); writing – review and editing (equal). **Sheng Lu:** Data curation (equal); investigation (equal); methodology (equal); writing – original draft (equal); writing – review and editing (equal). **Hongli Lv:** Project administration (lead); writing – original draft (equal); writing – review and editing (equal). **Xiujuan Wu:** Conceptualization (lead); funding acquisition (lead); writing – original draft (equal); writing – review and editing (equal).

## FUNDING INFORMATION

This study was supported by Xuhui District Medical Research Project (Grant no. SHXH202002) to Xiujuan Wu and Jiangsu University Research Project (Grant no. JDYY2023093) to Xiujuan Wu.

## CONFLICT OF INTEREST STATEMENT

The authors declare that they have no competing interests.

## Supporting information


**Figure S1.** Forest plot images. A causal association between B cell count and the risk of psoriasis vulgaris. (A) Absolute B cell count; (B) switched memory B cell count; (C) transitional absolute B cell count; (D) un‐switched memory B cell count. (a): memory B cell absolute count versus ‘non‐cancer illness code self‐reported: psoriasis’; (b) memory B cell absolute count versus ‘Psoriasis (vulgaris), strict definition’.

**Figure S2.** Funnel plot images. A causal association between B cell count and the risk of psoriasis vulgaris. (A) Absolute B cell count; (B) switched memory B cell count; (C) transitional absolute B cell count; (D) un‐switched memory B cell count. (a) Memory B cell absolute count versus ‘non‐cancer illness code self‐reported: psoriasis’; (b) memory B cell absolute count versus ‘Psoriasis (vulgaris), strict definition’.

**Figure S3.** LOO plot images. A causal association between B cell count and the risk of psoriasis vulgaris. (A) Absolute B cell count; (B) switched memory B cell count; (C) transitional absolute B cell count; (D) un‐switched memory B cell count. (a) Memory B cell absolute count versus ‘non‐cancer illness code self‐reported: psoriasis’; (b) memory B cell absolute count versus. ‘Psoriasis (vulgaris), strict definition’.

**Figure S4.** Scatter plot images. A causal association between B cell count and the risk of psoriasis vulgaris. (A) Absolute B cell count; (B) switched memory B cell count; (C) transitional absolute B cell count; (D) un‐switched memory B cell count. (a) Memory B cell absolute count versus ‘non‐cancer illness code self‐reported: psoriasis’; (b) memory B cell absolute count versus ‘Psoriasis (vulgaris), strict definition’.

## Data Availability

All data generated or analysed during this study are included in this article and supplementary information files.
