# Supplementary figures and images for: Causal association between B cell count and psoriasis using two‐sample Mendelian randomization
Source: J Cell Mol Med. 2024 Sep 11;28(17):e70089. doi: 10.1111/jcmm.70089 (PMC11390492; doi:10.1111/jcmm.70089)

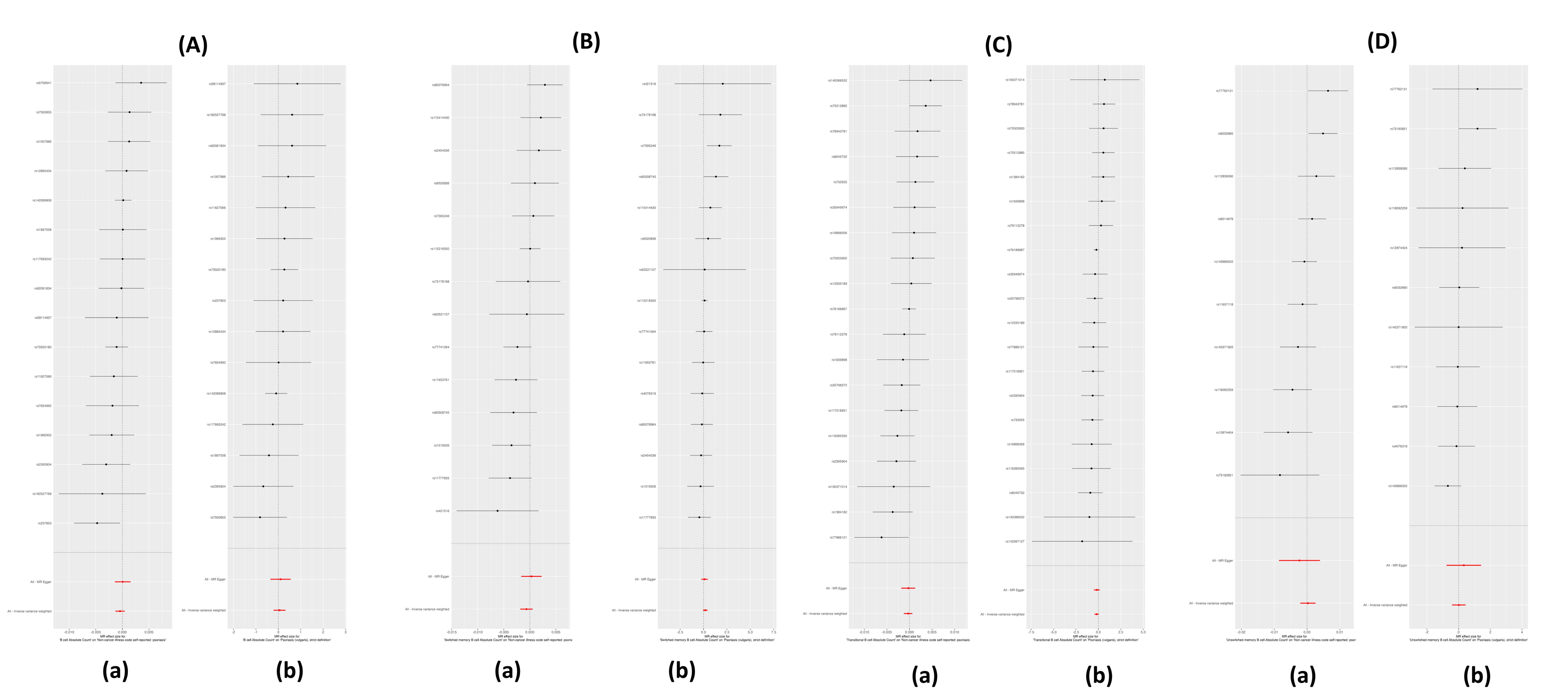

Supplement: Supplementary file 1 — Figure S1. Forest plot images. A causal association between B cell count and the risk of psoriasis vulgaris. (A) Absolute B cell count; (B) switched memory B cell count; (C) transitional absolute B cell count; (D) un‐switched memory B cell count. (a): memory B cell absolute count versus ‘non‐cancer illness code self‐reported: psoriasis’; (b) memory B cell absolute count versus ‘Psoriasis (vulgaris), strict definition’. [file JCMM-28-e70089-s001.jpg]

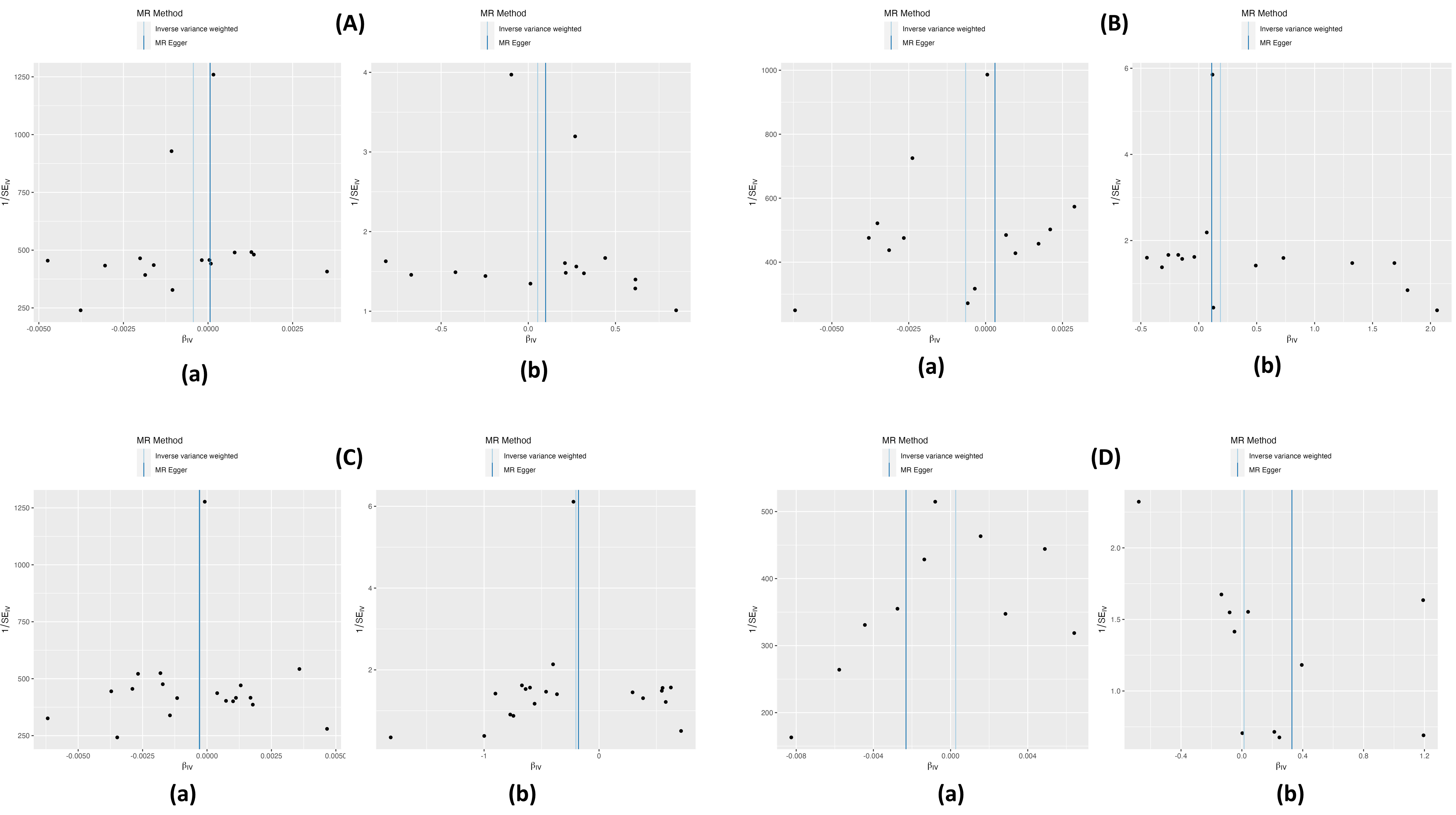

Supplement: Supplementary file 2 — Figure S2. Funnel plot images. A causal association between B cell count and the risk of psoriasis vulgaris. (A) Absolute B cell count; (B) switched memory B cell count; (C) transitional absolute B cell count; (D) un‐switched memory B cell count. (a) Memory B cell absolute count versus ‘non‐cancer illness code self‐reported: psoriasis’; (b) memory B cell absolute count versus ‘Psoriasis (vulgaris), strict definition’. [file JCMM-28-e70089-s002.jpg]

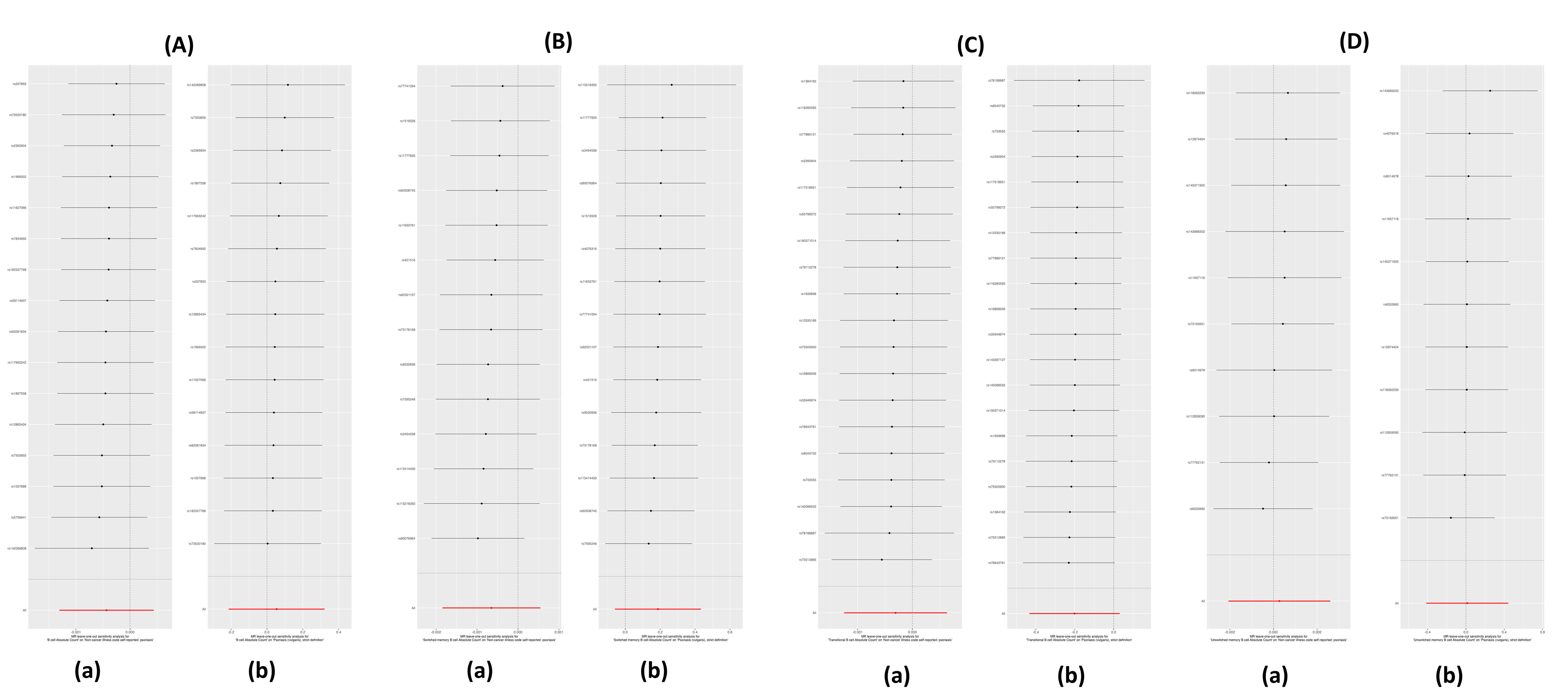

Supplement: Supplementary file 3 — Figure S3. LOO plot images. A causal association between B cell count and the risk of psoriasis vulgaris. (A) Absolute B cell count; (B) switched memory B cell count; (C) transitional absolute B cell count; (D) un‐switched memory B cell count. (a) Memory B cell absolute count versus ‘non‐cancer illness code self‐reported: psoriasis’; (b) memory B cell absolute count versus. ‘Psoriasis (vulgaris), strict definition’. [file JCMM-28-e70089-s003.jpg]

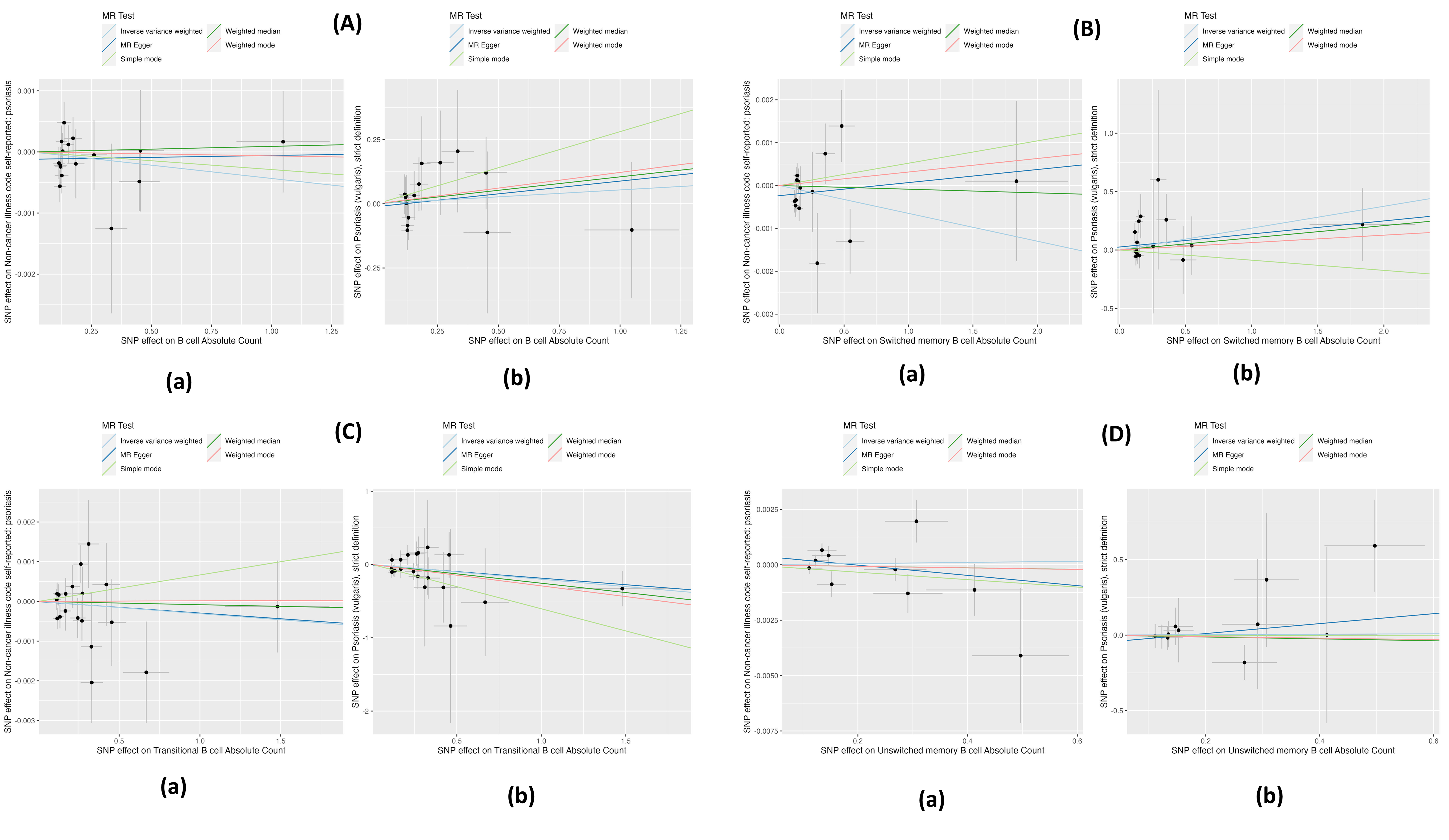

Supplement: Supplementary file 4 — Figure S4. Scatter plot images. A causal association between B cell count and the risk of psoriasis vulgaris. (A) Absolute B cell count; (B) switched memory B cell count; (C) transitional absolute B cell count; (D) un‐switched memory B cell count. (a) Memory B cell absolute count versus ‘non‐cancer illness code self‐reported: psoriasis’; (b) memory B cell absolute count versus ‘Psoriasis (vulgaris), strict definition’. [file JCMM-28-e70089-s004.jpg]
